# UPLC-MS-Based Metabolomics Profiling for α-Glucosidase Inhibiting Property of *Parkia speciosa* Pods

**DOI:** 10.3390/life11020078

**Published:** 2021-01-22

**Authors:** Mohammed S. M. Saleh, Juriyati Jalil, Nor Hidayah Mustafa, Fitri Fareez Ramli, Ahmad Yusof Asmadi, Yusof Kamisah

**Affiliations:** 1 Department of Pharmacology, Faculty of Medicine, Universiti Kebangsaan Malaysia, Cheras, Kuala Lumpur 56000, Malaysia; medsaleh@ukm.edu.my (M.S.M.S.); fitrifareez@ppukm.ukm.edu.my (F.F.R.); 2 Drug and Herbal Research Centre, Faculty of Pharmacy, Universiti Kebangsaan Malaysia, Kuala Lumpur 50300, Malaysia; juriyatijalil@ukm.edu.my (J.J.); norhidayahmustafa91@gmail.com (N.H.M.); 3 Unit of Pharmacology, Faculty of Medicine and Defence Health, Universiti Pertahanan Nasional Malaysia, Kem Sungai Besi, Kuala Lumpur 57000, Malaysia; draayusof@gmail.com

**Keywords:** α-glucosidase inhibitory activity, antidiabetic, flavonoids, phenolics, type 2 diabetes mellitus

## Abstract

*Parkia speciosa* is a food plant that grows indigenously in Southeast Asia. A great deal of interest has been paid to this plant due to its traditional uses in the treatment of several diseases. The pods contain many beneficial secondary metabolites with potential applications in medicine and cosmetics. However, studies on their phytochemical properties are still lacking. Therefore, the present study was undertaken to profile the bioactive compounds of *P. speciosa* pods collected from six different regions of Malaysia through ultra-high-performance liquid chromatography-quadrupole time-of-flight mass spectrometry (UHPLC-QTOF-MS) and α-glucosidase inhibitory potential. This study applied metabolomics to elucidate the differences between *P. speciosa* populations found naturally in the different locations and to characterize potential α-glucosidase inhibitors from *P. speciosa* pods. *P. speciosa* collected from different regions of Malaysia showed good α-glucosidase inhibitory activity, with a median inhibitory concentration (IC_50_) of 0.45–0.76 μg/mL. The samples from the northern and northeastern parts of Peninsular Malaysia showed the highest activity. Using UHPLC-QTOF-MS/MS analysis, 25 metabolites were identified in the pods of *P. speciosa*. The findings unveiled that the pods of *P. speciosa* collected from different locations exhibit different levels of α-glucosidase inhibitory activity. The pods are a natural source of potent antidiabetic bioactive compounds.

## 1. Introduction

Type 2 diabetes mellitus (T2DM) is a condition that is characterized by a high fasting plasma glucose level more than 7 mmol/L. It affects a large percentage of the population, with a global mortality rate of up to 4.9 million [1]. It often results in complications affecting the kidney, nervous system, vision, and heart [2]. T2DM is treated pharmacologically by using various drugs including α-glucosidase inhibitors such as acarbose, voglibose and miglitol [3]. α-Glucosidase is an enzyme that converts complex carbohydrate into digestible monosaccharides [4].

T2DM occurs due to pancreatic β-cells dysfunction, leading to insulin deficiency [5]. It is believed that reactive oxygen species (ROS) contribute to the loss of pancreatic β-cell function and survival [6,7]. Therefore, it is anticipated that natural bioactive products with antioxidant properties will decrease the detrimental effects of ROS on pancreatic β-cells, and improve diabetes mellitus. Many studies have shown the hypoglycemic effects of natural products on T2DM, one of which is *Parkia speciosa* Hassk extract [8]. In these studies, α-glucosidase was employed as the target enzyme [4,9]. A great deal of attention has been directed towards medicinal plants with antioxidant or enzyme inhibiting properties for their potential uses as safer alternatives for disease treatment [10]. 

*P. speciosa*, a rainforest tree, belongs to the family Fabaceae [11]. The plant thrives on podzolic sandy loam and in areas nearby riverbanks in primary lowland rainforests in Southeast Asia. It is a perennial plant that grows up to 40 m tall and 1 m in stem circumference. Its leaves are bipinnate and alternate. It starts to flower and produce fruit at the age of seven years. It usually flowers in January to March and August to October each year. The flowers are bulb-shaped and droop at the stalk ends, while the fruits are green, flat and oblong pods of 35–55 cm in length and 3–5 cm in width, beetling in bunches of 6–10. Each pod contains 10–18 bright green almond-shaped seeds [12]. Each tree can produce an average of 250–300 pods per season [13]. Its seeds are consumed with or without the pods, either raw or cooked. The plant has been used as a traditional medicine in several parts of Southeast and South Asia to treat dermatitis, toothache, kidney disorder, diabetes, hypertension, malaria, skin problems, and diarrhea [13]. The pods possess anti-inflammatory, anticancer, antimicrobial, antioxidant, and antidiabetic properties [14]. These activities have been linked to the presence of various secondary metabolites in this perennial plant, including phenolic acids, tannins, flavonoids, triterpenes, and polysaccharides [11,15,16]. With the identification of several bioactive compounds in *P. speciosa*, the plant has a high value for development as a nutraceutical. Previous studies have reported the profiles and compositions of metabolites that are present in the pods [10,15]. It is known that different geographical regions could affect the composition and quality of the plants [17]. Therefore, the aim of this study was to characterize *P. speciosa* pod profiles, sampled from different regions in Malaysia using a highly sensitive metabolomics-based ultra-high-performance liquid chromatography-quadrupole time of flight/tandem mass spectrometry (UPLC-ESI-QTOF/MS) and α-glucosidase assay.

## 2. Materials and Methods

### 2.1. Chemical and Reagents

α-Glucosidase from *Saccharomyces cerevisiae* (lyophilized powder), *p*-nitrophenyl-α-D-glucopyranoside (*p*-NPG, ≥98%.), chromatographic-grade formic acid and methanol were purchased from Fisher Scientific (Waltham, MA, USA). Other analytical grade chemicals used were purchased from Merck (Darmstadt, Germany).

### 2.2. Plant Collection

**Figure 1:**
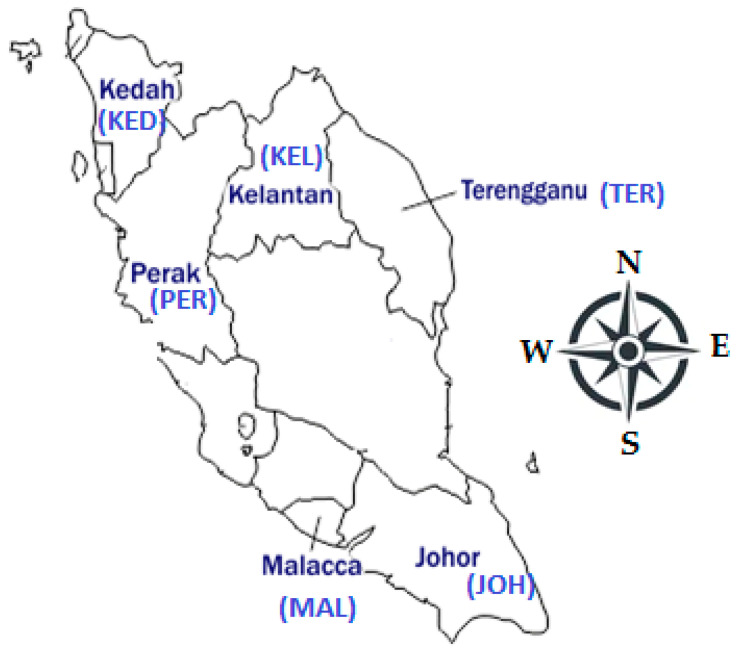
Location map showing the plant collection sites.

*P. speciosa* pods were collected from six different locations (Figure 1) of Peninsular Malaysia—Terengganu (TER; eastern part), Johor (JOH; southern part), Kelantan (KEL; northeastern part), Malacca (MAL; southwestern part), Perak (PER; western part), and Kedah (KED; northern part) in February 2020. The collected plants were identified, and a voucher specimen (ID009/2020) was deposited at the Universiti Kebangsaan Malaysia Herbarium.

### 2.3. Sample Drying and Extraction

Fresh *P. speciosa* deseeded pods were rinsed with water and cleaned with tissue paper. The pods were peeled, cut into small pieces, and placed at −80 °C to prevent degradation or loss of secondary metabolites before being subjected to freeze-drying for 5 days. Then, the samples were ground finely and sieved to 60 mesh size particles. The resultant powder was stored in an airtight bag at −80 °C. A volume of 200 mL of ethanol was added to 20 g of the powder and subjected to extraction by sonication (320 W, 30 °C, 1 h). The solution was centrifuged for 5 min at 10,000 rpm and passed through a Whatman filter paper to obtain the supernatant. The remaining ethanol was removed with a rotary vacuum evaporator at 40 °C. Finally, the extracts were dried in a freeze-dryer and kept at −80 °C until further use.

### 2.4. Estimation of α-Glucosidase Inhibitory Activity

α-Glucosidase inhibitory activity of *P. speciosa* extracts was examined following a previously described method with a slight modification [18]. A stock solution of the substrate *p*-NPG (0.3 mg/mL) was prepared in 50 mM phosphate buffer (pH 6.5). Equal volumes (10 µL) of diluted crude extracts in various concentrations (0.2–2.0 mg/mL) and α-glucosidase (2 U/µL in 50 mM phosphate buffer) were added into 100 μL of phosphate buffer (30 mM) in a 96-well plate. The mixture was incubated for 5 min at room temperature, after which 75 μL of *p*-NPG solution was added. The mixture was then incubated for 30 min before termination of reaction by the addition of 50 μL of glycine solution (2 M), and the absorbance was measured at 405 nm. The activity determination in the samples was carried out six times for each extract. The median inhibitory concentration (IC_50_) for each extract was obtained. Quercetin was used as the standard α-glucosidase inhibitor [19].

### 2.5. UHPLC-QTOF-MS

The analyses of bioactive compounds present in *P. speciosa* extracts were performed using Agilent 1290 Infinity II UHPLC System (Santa Clara, CA, USA) and 6550 iFunnel Q-TOF LC/MS system (Agilent Technologies, Santa Clara, CA, USA). Separation was carried out using a C18 column (100 mm × 2.1 mm I.D.; 3 μm particle size) (Agilent Technologies, Santa Clara, CA, USA). The mobile phase consisted of 0.1% formic acid in water (solvent A) and methanol (solvent B) at a flow rate of 0.4 mL/min at 25 °C. The elution conditions were: 0 min, 85% A; 2–10 min, 50% A; 15 min, 30% A; 20 min, 15% A; 22–23 min, 0% A; 26 min, 85% A. MS detection was carried out in negative ion mode using a mass range of 100–1200 m/z. The negative ion mode was chosen because it appeared more selective and more sensitive for further LC-MS analysis of flavonoids and phenolics in plants. The extracted samples were dissolved in methanol, thoroughly mixed, filtered with a nylon filter of 0.45 μm and analyzed.

### 2.6. Multivariate Data Analysis

Multivariate data analysis (MVDA) was conducted according to the method by Abd Ghafar et al. [20] with a slight modification. UHPLC-QTOF-MS chromatograms in negative ion mode were used for untargeted analysis. The raw data acquired by Xcalibur 2.2 (Thermo Fisher Scientific, Waltham, MA, USA) were first converted into mzXML files using the MSConvert software [21], subsequently, the data were pre-treated using MZmine [22]. The software allowed noise from LC-QTOF-MS profiles to be removed (Noise level 5.0 E3), and data points under this intensity to be excluded from analysis. A number of 1993 peak mass signals were identified. The processed data were exported as a .cvs file (table format). The peak areas derived from LC-QTOF-MS analysis were further analyzed using principal components analysis (PCA) with UV scaling applied via SIMCA^®^ P+ software 14.0 (Umetrix AB, Umea Sweden), prior to MVDA to normalize the data. Partial least squares (PLS) regression was employed to study the relationship between the two data sets by performing discriminant classification of different types of samples. Numeric values (+2, +1, −1 or −2) were assigned to four classes, followed by targeted analysis. To prepare the data matrix, raw data extracted from UHPLC-QTOF-MS were used to prepare a table containing the areas obtained from each sample. The dataset table obtained showed the area of each marker compound in the rows, and the different samples in the columns for the variables and observations, respectively. This was used as the X component. The data matrix table included data on α-glucosidase inhibitory activity, retrieved from the test results and expressed as IC_50_. This was used as the Y component to perform PCA as an investigatory analysis and PLS. The results provided insight into correlations between the dataset and α-glucosidase inhibition activities.

### 2.7. Statistical Analysis

The data were reported as means ± standard deviation of six biological replicates. Statistical analysis was performed via a one-way ANOVA followed by Tukey post hoc test with Minitab 17 (Minitab Inc., State Collage, PA, USA). Significant differences were considered at 95% confidence level.

## 3. Results

### 3.1. α-Glucosidase Inhibitory Activity

The yields of *P. speciosa* pod extracts sampled from different locations were similar. The extracts effectively inhibited α-glucosidase activity with IC_50_ values ranging from 0.443 to 0.707 µg/mL, which were more potent than the quercetin (3.550 μg/mL) (Table 1). *P. speciosa* extracts presented over 95% α-glucosidase inhibition at 2 μg/mL concentration in a dose-dependent manner (data not shown). The extracts of KEL had the highest inhibitory activity, followed by KED, PER, MAL, TER, and JOH being the lowest.

### 3.2. UHPLC-QTOF-MS/MS Identification

**Figure 2:**
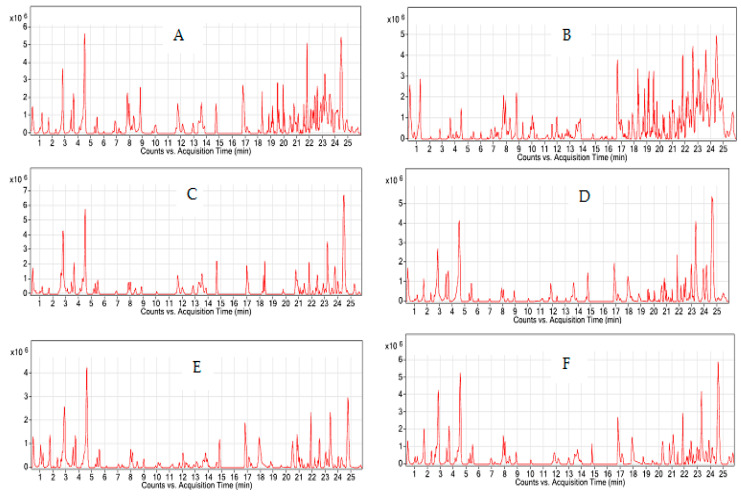
Total ion chromatogram of *P. speciosa* pod extracts collected from different locations in Malaysia: (**A**) TER, Terengganu; (**B**) JOH, Johor; (**C**) KEL, Kelantan; (**D**) MAL, Malacca; (**E**) PER, Perak; (**F**) KED, Kedah.

Comparisons based on available databases, including the Human Metabolome Database (HMDB), Metlin, KnaPSacK, MassBank, Kyoto Encyclopedia of Genes and Genomes (KEGG), and PlantCyc resulted in the tentative identification of 25 metabolites in the extracts based on the retention times. Mass, fragmentation pathways and MS/MS spectral data were compared against the reference database (Supplementary Table S1). Total ion chromatogram (TIC) data were also obtained during the analysis (Figure 2). There was a marked intensity variation between the samples collected from TER and JOH with those from other locations, particularly in flavonoids. Table 2 shows the predicted identities of the compounds in the extracts from different locations. Retention time, molecular ion and fragmentation data of each compound were also reported. Some of the compounds had been reported in previous studies, new compounds were also identified including gallocatechin-(4α→8)-epigallocatechin, epigallocatechin, theasinensin A, epigallocatechin gallate (three isomers), gossypetin 8-glucoside, gossypetin 8-rhamnoside (4 isomers), myricitrin, eucommin A, 6-c-galactosylluteolin (two isomers), curcumin monoglucoside, herbacetin, tremulacin, 2-phenylethanol glucuronide, and embelin. However, some of these metabolites were absent in some of the locations, for example, theasinensin A and theaflavin-3-gallate were absent from the samples collected from JOH, while samples obtained from KEL lacked 6-c-galactosylluteolin. MS/MS analysis of the extracts indicated that herbacetin and 2-phenylethanol glucuronide were only present in the JOH samples, while epigallocatechin and curcumin monoglucoside were only found in the TER samples.

### 3.3. Multivariate data analysis (MVDA)

**Figure 3:**
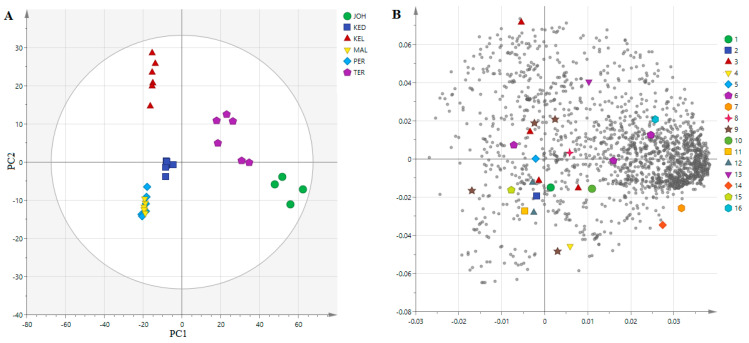
The principal components analysis (PCA) score plot (**A**) and the loading plot (**B**) of the MS data in the pods of *P. speciosa* collected from different locations in Malaysia. 1. gallic acid; 2. gallocatechin-(4α→8)-epigallocatechin; 3. theaflavin-3-gallate; 4. epigallocatechin; 5. theasinensin A; 6. epigallocatechin gallate isomers; 7. 2-phenylethanol glucuronide; 8. gossypetin 8-glucoside isomers; 9. gossypetin 8-rhamnoside isomers; 10. myricitrin; 11. eucommin A; 12. 6-c-galactosylluteolin; 13. curcumin monoglucoside; 14. herbacetin; 15. tremulacin; 16. embelin.

The 1993 mass signals extracted from the UHPLC-QTOF-MS results were analyzed using MVDA and organized as a score plot and a loading plot for principal component 1 (PC1) and 2 (PC2) (Figure 3). This model can be explained by six PC. The score plot, PC1 and PC2 showed total variance of 47.8% with PC1 38.5%, and PC2 9.3%, separating the six different locations (TER, KED, PER, MAL, JOH, and KEL). The PCA score plot showed the separation of four different clusters (Figure 3A). The location of *P. speciosa* sampling was the main factor for the separation of the principal components. Meanwhile, the loading plot allowed for us to identify metabolites in *P. speciosa* from different locations by observing the mass signals. 

The loading plot (Figure 3B) showed the effect of different locations of *P. speciosa* on the composition of metabolites, including gallic acid, gallocatechin-(4α→8)-epigallocatechin, theaflavin-3-gallate isomers, epigallocatechin, theasinensin A, epigallocatechin gallate isomers, gossypetin 8-glucoside isomers, gossypetin 8-rhamnoside isomers, myricitrin, eucommin A, 6-c-galactosylluteolin, curcumin monoglucoside, herbacetin, tremulacin, and embelin. The loading plot showed that the metabolite contents were higher in the samples attained from the JOH and TER.

### 3.4. PLS Analysis of UHPLC-MS Data

**Figure 4:**
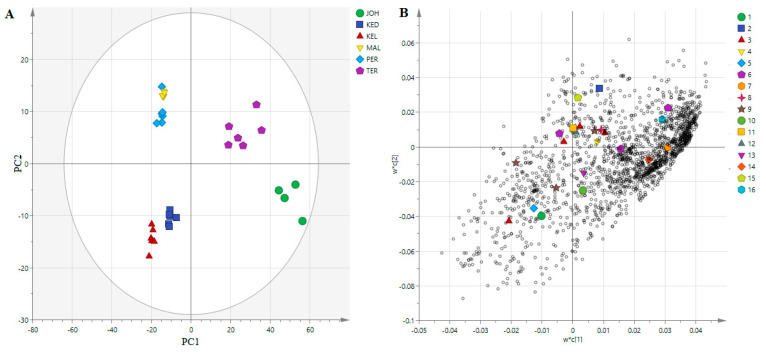
The partial least squares (PLS) regression score plot (**A**) and the loading plot (**B**) of the MS data in the *P. peciosa* pods obtained from different locations in Malaysia. 1. gallic acid; 2. gallocatechin-(4α→8)-epigallocatechin; 3. theaflavin-3-gallate; 4. epigallocatechin; 5. theasinensin A; 6. epigallocatechin gallate isomers; 7. 2-phenylethanol glucuronide; 8. gossypetin 8-glucoside isomers; 9. gossypetin 8-rhamnoside isomers; 10. myricitrin; 11. eucommin A; 12. 6-c-galactosylluteolin; 13. curcumin monoglucoside; 14. herbacetin; 15. tremulacin; 16. embelin.

**Figure 5:**
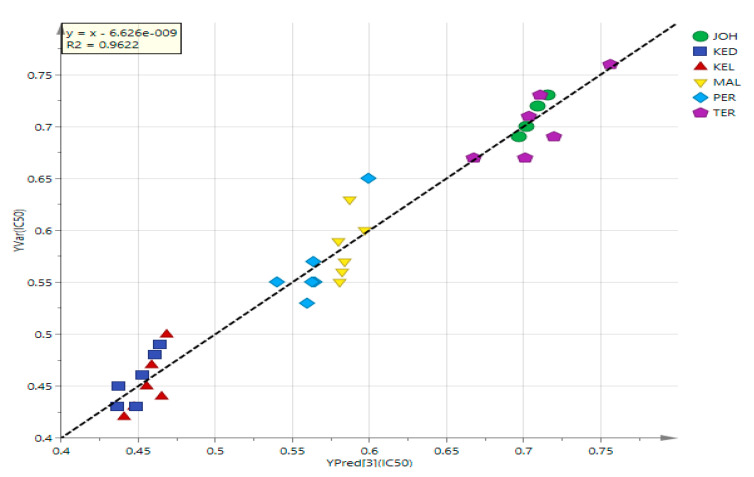
Partial least squares (PLS) regression plot line loading of *P. speciosa* pod extracts.

**Figure 6:**
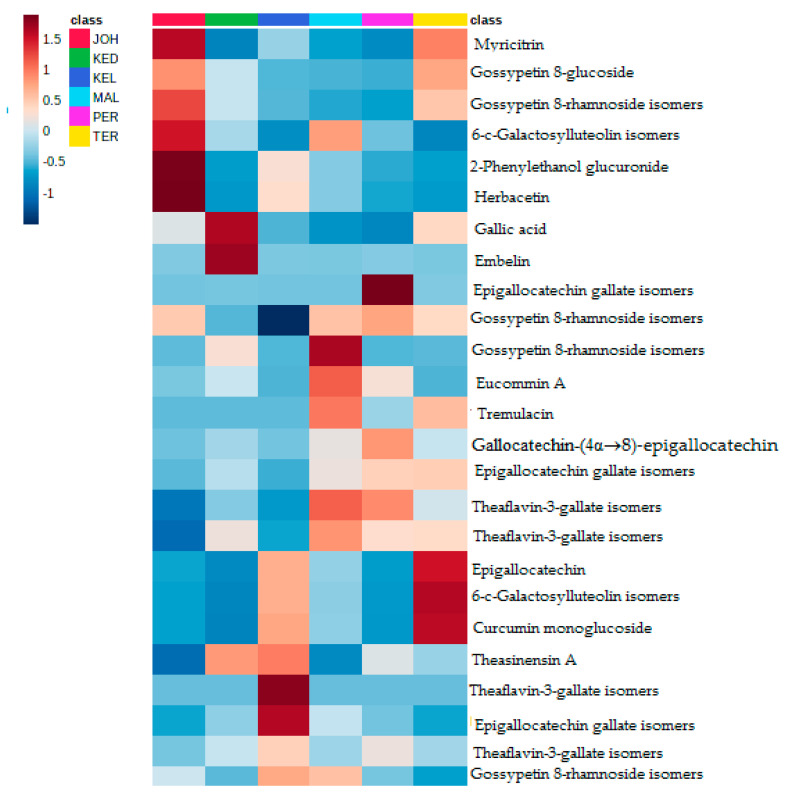
Heatmap of identified bioactive compounds of *P. speciosa* pods. Color indicates metabolites expression value: absent (blue) to present (brown).

PLS analysis was performed on MS and α-glucosidase inhibitory data to determine the relationship between the two variables (X and Y) (Figure 4A), to see the closeness of the extracts on the plot area, as well as to identify variables with the highest effects on the α-glucosidase inhibitory activity. Similar to the PCA loading plot, the contents of metabolites from the JOH and TER were bigger than from other locations (Figure 4B). This model plot showed an R^2^ of 0.9622, which indicated that the model functioned considerably well (Figure 5). The heatmap, an unsupervised clustering, was constructed based on the identified 25 biomarkers. The heatmap showed that the metabolites had a differential distribution between the six locations (Figure 6).

## 4. Discussion

In the current study, *P. speciosa* was investigated for its ethnomedicinal use as a glucose-lowering agent. The plant contains metabolites including phenols, fatty acids, alkaloids and steroids that confer various biological activities [23]. The most common and important compounds found in the plant are polyphenols, in particular, flavonoids [16,24,25]. Dietary intake of polyphenols, including flavonoids, is reported to be beneficial in terms of conferring insulin resistance and is associated with a lower risk of T2DM in humans [26,27]. Additionally, the flavonoid glycosides, flavone and phenolic acid, were demonstrated to exhibit various pharmacological activities including anticancer, antioxidant, anti-inflammatory, and cardioprotective activities [28,29].

This is the first study to report on the phytochemical profiles and screening of α-glucosidase-inhibiting compounds in *P. speciosa* pods using UPLC-QTOF-MS/MS. A total of 25 compounds were identified comprising 19 flavonoids, three phenolic acids and three other compounds. Eight flavonoid glycosides and 11 flavanols were identified in the extracts. A total of 24 metabolites were reported for the first time in *P. speciosa* pods. However, epigallocatechin and epigallocatechin gallate were reported in different species of *Parkia* [13], while gallic acid was previously identified in the pods of *P. speciosa* [14]. 

Most of these compounds have been reported for their ability to inhibit *α*-glucosidase activity [30]. These metabolites are most likely to be responsible for the α-glucosidase inhibitory activity of the plant. Moreover, the flavanol compounds (epigallocatechin, epigallocatechin gallate isomers, theasinensin A, theaflavin-3-gallate isomers, gallocatechin-(4α→8)-epigallocatechin and herbacetin) identified in *P. speciosa* most likely have a major role in the α-glucosidase inhibitory activity due to the structural configuration of double bonds conjugated to 4-oxo and hydroxyl groups [30]. It is quite hard to specifically pinpoint the most likely metabolites that were responsible for the high α-glucosidase-inhibiting property in the samples attained from KED and KEL, since almost all metabolites present in the pods from the locations were also detected in other locations. It is possible that samples originating from both KED and KEL had higher concentrations of the α-glucosidase-inhibiting compounds. However, this could not be confirmed since the metabolites were only analyzed qualitatively. Even though the JOH and TER samples contained more metabolites, their α-glucosidase-inhibiting activities were lower compared to the other four locations, suggesting that not all metabolites present in these samples (JOH and TER) contributed to the activity. While the pods from KEL had the least flavonoid contents—theaflavin-3-gallate, theasinensin A, epigallocatechin gallate and gossypetin 8-rhamnoside—compared to other locations but were better in inhibiting α-glucosidase. However, the activities in samples from the six locations were significantly more potent than that of quercetin, the positive control. These findings are consistent with the PLS regression results, suggesting that these metabolites may assist the clustering of metabolites and could be responsible for the α-glucosidase inhibitory activities of the pods. 

A previous study reported that myricitrin had a significantly higher α-glucosidase inhibitory activity (IC_50_ = 8.65 μg/mL) than quercetin [31], suggestive that the substitution of sugar units or hydroxyl group with galloyl increased the efficacy of flavonoids against *α*-glucosidase. Our results are in accordance with previous studies reporting high α-glucosidase inhibitory activity in plant extracts such as *Phyllanthus acidus* [32], *Potentilla inclinata* [33], *Tetracera macrophylla* [34], *Ligustrum vulgare* L [35] and *Tetracera indica* [36] owing to the presence of phenolic acids and flavonoids. The findings obtained in the current study suggested that *P. speciosa* pod extracts could be used to alleviate postprandial hyperglycemia.

Metabolomics profiling of *P. speciosa* samples collected from the six different regions of Malaysia showed a significant difference between the samples in terms of the total content of investigated compounds. The data expressed by PCA and those proposed by the loading plot are interrelated and complementary to each other. On the one hand, this result clearly indicates that this separation is the result of the dissimilarity in phytochemical contents between the six extracts, and on the other hand, it reveals that the phytochemical contents of MAL, KED and PER are very similar, due to the existence of the same metabolites with different magnitudes. Samples collected from the JOH and TER regions indicated the lowest flavonoid contents. Many climatic or abiotic factors such as temperature, rainfall, humidity, as well as solar radiation can affect the biosynthesis of secondary metabolites in plants [27]. The age of the plants, watering patterns and types of soil could also influence the phytochemical content and composition. Unfortunately, these factors could not be ascertained in the current study since the samples were not collected from cultivated plants, but from plants that grew in the wild. A recent study on some plant extracts also demonstrated that geographical location had a significant effect on the composition and content of secondary metabolites [17,27]. This could explain the differences noted on the metabolites detected in pod samples collected from different locations in the current study and their intensities that are shown in the heatmap. Herbacetin and 2-phenylethanol glucuronide were unique to samples from JOH, which lacked theaflavin-3-gallate, epigallocathechin, gallocatechin-(4α→8)-epigallocatechin, and theasinensin A. These metabolites (herbacetin and 2-phenylethanol glucuronide) could be chemical markers for pods obtained from JOH, while epigallocatechin and curcumin monoglucoside could be markers for pods found in TER. The phytoconstituents of the pods from KEL and KED should be investigated further to better understand their α-glucosidase inhibitory activity. These findings are suggestive that the pods from both locations are better natural sources to isolate potential α-glucosidase inhibitors.

## 5. Conclusions

*P. speciosa* pods sampled from different parts of Malaysia displayed dissimilar α-glucosidase inhibitory activities which correlated with their metabolites, which were characterized by UHPLC-QTOF chemical profiling indicating the presence of flavonoids and phenolic compounds in the pod extracts. Hence, *P. speciosa* pods have the potential to be developed as an α-glucosidase inhibitor.

## Figures and Tables

**Table 1:** Yield and α-glucosidase inhibitory activity of *P. speciosa* pods collected from different locations of Peninsular Malaysia.

Location	Yield (%)	α-Glucosidase Inhibitory Activity (IC_50_) (µg/mL)
TER	16.497 ± 0.702 ^a^	0.705 ± 0.036 ^b^
JOH	17.393 ± 0.632 ^a^	0.707 ± 0.016 ^b^
KEL	16.833 ± 1.048 ^a^	0.443 ± 0.036 ^d^
MAL	16.700 ± 0.605 ^a^	0.583 ± 0.029 ^c^
PER	16.087 ± 0.784 ^a^	0.567 ± 0.043 ^c^
KED	16.875 ± 0.996 ^a^	0.457 ± 0.025 ^d^
Quercetin	-	3.550 ± 0.130 ^a^

Values represent mean ± SD (*n* = 6). The values that do not share a same letter (^a–d^) are significantly different (*p* < 0.05). Abbreviations: Terengganu (TER; eastern part), Johor (JOH; southern part), Kelantan (KEL; northeastern part), Malacca (MAL; southwestern part), Perak (PER; western part), and Kedah (KED; northern part).

**Table 2:** Metabolites identified from *P. speciosa* pods sampled from six different locations by ultra-high-performance liquid chromatography-quadrupole time-of-flight mass spectrometry (UPLC-QTOF-MS/MS).

No.	RT (min)	[M–H]^−^ (m/z)	m/z	MW	Formula	MS/MS	ppm	Tentative Identification	TER	JOH	KEL	MAL	PER	KED
1	1.196	169.0142	170.0219	170.1200	C_7_ H6 O_5_	125.0245, 107.0132	−2.23	Gallic acid	+	+	-	-	-	+
2	1.705	609.1249	610.1295		C_30_ H_26_ O_14_	483.0907, 441.0813, 423.0708, 305.0658, 303.0506, 273.0417, 177.0192, 137.0258, 125.0244, 137.0258	−0.52	Gallocatechin-(4α→8)- epigallocatechin	+	-	-	+	+	+
3	2.298	760.6424	716.1379	716.6040	C_36_ H_28_ O_16_	609.1227,591.1129, 423.0714, 305.0657	−2.2	Theaflavin-3-gallate isomers	-	-	-	+	+	+
	2.705						1.67		+	-	+	+	+	+
	2.891						−2.18		-	-	-	+	+	+
	5.246						−1.93		+	-	+	+	+	+
4	3.419	457.0785	458.372	458.3720	C_15_ H_14_ O_7_	331.0458, 305.0666, 287.0561, 269.0463, 193.0146, 169.0152, 125.0254	−1.72	Epigallocatechin	+	-	-	-	-	-
5	3.594	912.6556	914.1546	914.7000	C_44_ H_34_ O_22_	761.1333, 743.1231, 591.1131M 573.1024, 423.0714, 177.0198	−0.02	Theasinensin A	+	-	+	+	+	+
6	4.017	457.0778	458.0841	458.3720	C_22_ H_18_ O_11_	331.0458, 305.0665, 287.0558, 269.0471, 193.0144, 169.0148, 125.0255	−1.48	Epigallocatechin gallate isomers	+	-	-	+	+	+
	4.305						−2.96		-	-	+	+	+	+
	4.505						−3.18		+	+	-	+	+	+
7	4.139	343.1032 + (HCOO)-	298.29	298.1050	C_14_H_18_O_7_	253.073, 223.0601, 208.0385, 206.021, 195.0657181.0497, 177.02		2-Phenylethanol glucuronide	-	+	-	-	-	-
8	6.836	479.0814	480.0946	480.4000	C_21_H_20_O_13_	461.0666, 316.0213, 287.0178, 271.0245, 243.0276, 214.0279, 179.0002, 151.0034, 124.0171	1.65	Gossypetin 8-glucoside	+	+	-	-	-	+
9	6.748	463.0857	464.096	464.4000	C_21_ H_20_ O_12_	343.0425, 301.0335, 271.0243, 255.0283, 243.0261, 178.9978, 151.002	1.14	Gossypetin 8-rhamnoside isomers	+	+	-	-	-	+
	7.104						0.67		+	+	-	-	+	+
	7.04						0.64		+	+	-	+	-	+
	7.688						0.49		+	+	+	+	+	+
10	7.955	463.0882	464.0961	464.3700	C_21_ H_20_ O_12_	445.0769, 445.0769, 343.0423, 300.0274, 271.0243, 255.0298, 243.0298, 178.9985, 151.0045	−1.24	Myricitrin	+	+	-	-	-	-
11	8.345	595.2027 + (HCOO)-	550.6020	550.6000	C_27_ H_34_ O_12_	595.2022, 475.1579, 431.1332, 369.114, 329.1004, 311.0916, 205.0503, 190.0283	1.16	Eucommin A	-	+	-	+	+	+
12	8.408	448.1012	447.0940	447.4000	C_21_ H_20_ O_11_	301.0349, 272.0317, 255.0297, 243.0291, 227.0349, 151.0058, 105.0203	0.56	6-c-Galactosylluteolin isomers	+	-	-	-	-	-
	8.76													
							−0.7		-	+	-	+	+	+
13	10.068	75.1763 + (M + HCOO)	530.1778	530.5000	C_27_ H_30_ O_11_	559.1445, 279.9182, 170.8336	1.92	Curcumin monoglucoside	+	-	-	-	-	-
14	10.088	301.0351	302.0426	302.23OO	C_15_H_10_O_7_	273.0399, 257.0437, 245.0428, 192.0079, 178.9975, 164.0087, 151.0037, 121.0286, 107.0143	0.19	Herbacetin	-	+	-	-	-	-
15	11.042	573.1608 + (M + HCOO)^−^	528.1632	528.5000	C_27_ H_28_ O_11_	409.0912, 325.0684, 307.0597, 283.0623, 117.0364	1.92	Tremulacin	+	-	-	+	+	-
16	13.858	293.1753	294.1828	294.4000	C_17_ H_26_ O_4_	263.1631, 257.1775, 237.1073, 235.1324, 221.1541, 190.1001	0.16	Embelin	-	+	-	-	+	+

Abbreviations: TER: Terengganu; JOH: Johor; KEL: Kelantan; MAL: Malacca; PER: Perak; KED: Kedah; +: present; -: absent.

## Data Availability

The data presented in this study are available on request from the corresponding author.

## Supplementary Materials

The following are available online at https://www.mdpi.com/2075-1729/11/2/78/s1, Figure S1. MS/MS fragmentations spectra of Gossypetin 8-rhamnoside, Eucommin A, E mbelin, and gallic acid.
