# Supplementary material for: UPLC-MS-Based Metabolomics Profiling for α-Glucosidase Inhibiting Property of Parkia speciosa Pods
Source: Life (Basel). 2021 Jan 22;11(2):78. doi: 10.3390/life11020078 (PMC7910992; doi:10.3390/life11020078)
Supplement: Supplementary file 1 [file life-11-00078-s001.pdf]

Figure. S1. MS/MS fragmentations spectra of Gossypetin 8-rhamnoside, Eucommin A, E mbelin, and gallic acid

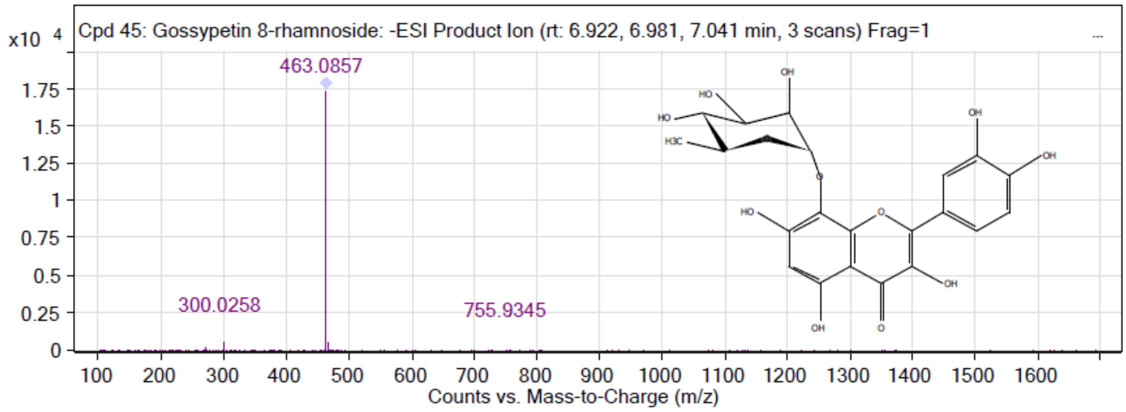

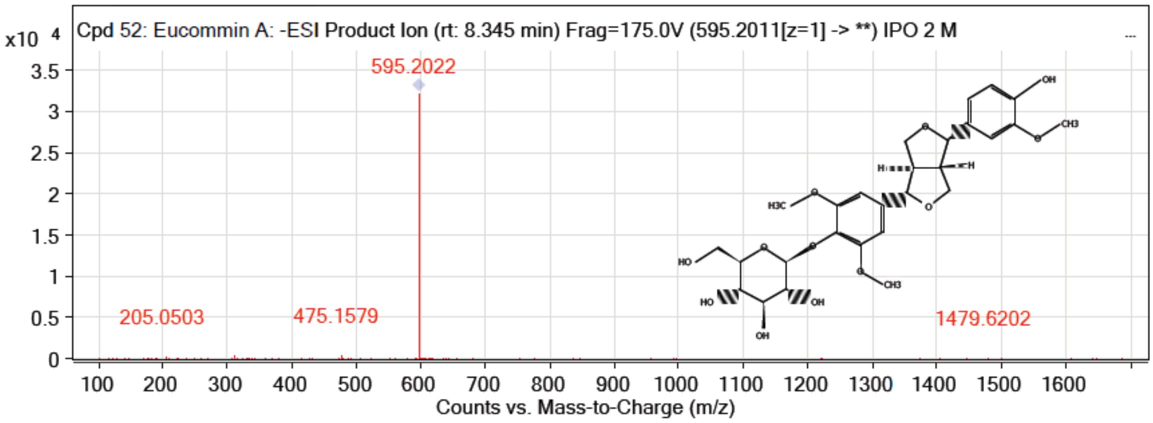

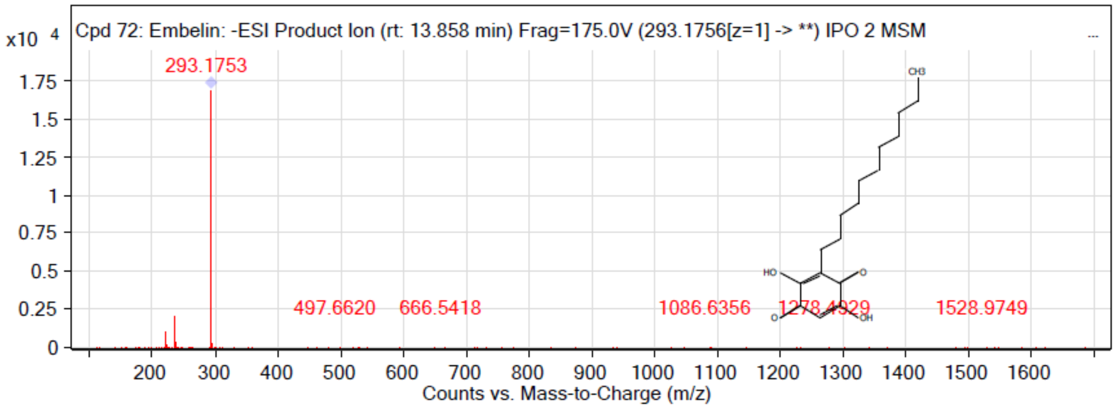

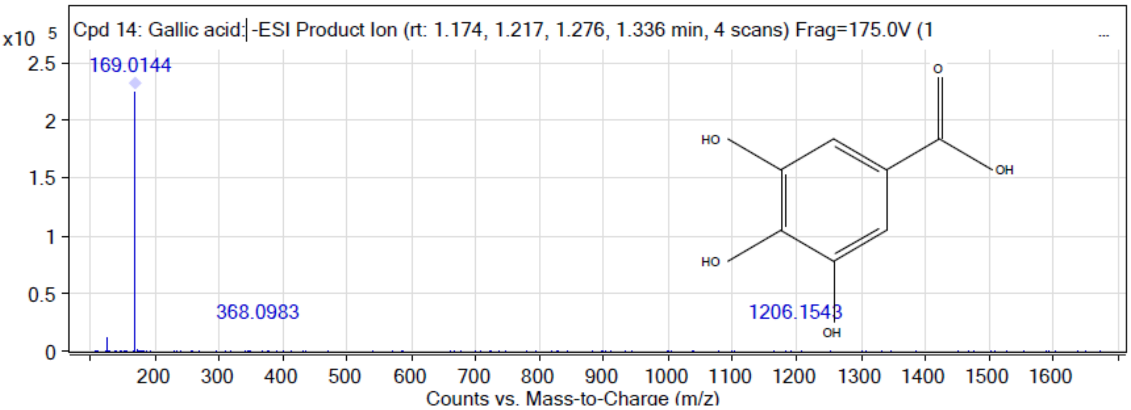

x10 5
Cpd 14: Gallic acid: -ESI Product Ion (rt: 1.174, 1.217, 1.276, 1.336 min, 4 scans) Frag=175.0V (1
169.0144
368.0983
1206.1543
HO
HO
OH
O
OH
0
0.5
1
1.5
2
2.5
200 300 400 500 600 700 800 900 1000 1100 1200 1300 1400 1500 1600
Counts vs. Mass-to-Charge (m/z)
